# Fatal Infectious Disease Surveillance in a Medical Examiner Database^1^

**DOI:** 10.3201/eid1001.020764

**Published:** 2004-01

**Authors:** Mitchell I. Wolfe, Kurt B. Nolte, Steven S. Yoon

**Affiliations:** * Centers for Disease Control and Prevention, Atlanta, Georgia, USA; † Office of the Medical Investigator, University of New Mexico School of Medicine, Albuquerque, New Mexico, USA

**Keywords:** medical informatics applications, algorithms, sentinel surveillance, emerging communicable diseases, forensic medicine, coroners and medical examiners

## Abstract

Increasing infectious disease deaths, the emergence of new infections, and bioterrorism have made surveillance for infectious diseases a public health concern. Medical examiners and coroners certify approximately 20% of all deaths that occur within the United States and can be a key source of information regarding infectious disease deaths. We hypothesized that a computer-assisted search tool (algorithm) could detect infectious disease deaths from a medical examiner database, thereby reducing the time and resources required to perform such surveillance manually. We developed two algorithms, applied them to a medical examiner database, and verified the cases identified against the opinion of a panel of experts. The algorithms detected deaths with infectious components with sensitivities from 67% to 94%, and predictive value positives ranging from 8% to 49%. Algorithms can be useful for surveillance in medical examiner offices that have limited resources or for conducting surveillance across medical examiner jurisdictions.

 Infectious disease deaths in the United States substantially declined during the first 8decades of the 20th century as a result of public health interventions. However, the end of the century was marked by an increase in infectious disease deaths primarily due to AIDS and pneumonia and influenza (*1*,*2*). Increasing infectious disease deaths, the emergence of new infections, and the real or perceived threat of bioterrorist activities have made surveillance for infectious diseases a public health need (*3*,*4*).

Infectious disease mortality trends have been described by review of International Classification of Diseases (ICD)*–*coded death certificate data (*2*). Although useful in identifying trends, this process has certain limitations, including the following: causes of death are inaccurately certified, are not autopsy verified, and are erroneously coded; and ICD codes are not arranged to facilitate aggregation of infectious disease mortality data or designed to identify new infectious diseases (*5*). Medical examiners and coroners are also a source of surveillance data for infectious disease deaths. These investigators certify (i.e. enter information about the cause and manner of death on death certificates) approximately 20% of all deaths that occur within the United States (*6*). Medicolegal death investigation systems are often biased towards the investigation of violent or unnatural deaths. However, sudden natural deaths, unexplained deaths, and deaths of public health importance are also investigated by these agencies (*5*,*7*–*11*).

Natural disease deaths investigated by medical examiners and coroners are often caused by infectious processes (*12*). Additionally, their investigation frequently includes a complete autopsy. In recent years, medical examiners and coroners have recognized outbreaks of hantavirus pulmonary syndrome and invasive pneumococcal disease, identified cases of human plague, and participated in the investigation of West Nile encephalitis (*13*–*16*). In the 2001 outbreak of bioterrorism-related anthrax, all the deaths were investigated by medical examiners (*9*–*11*,*17*). Consequently, medical examiner/coroner databases can be a key source of information about infectious diseases, both in outbreak and nonoutbreak settings.

In general, medical examiners are appointed physician pathologists, usually with special training in performing forensic autopsies and medicolegal death investigations; coroners are usually elected officials, may not be physicians, and rely on other medical personnel for death investigation and autopsy services (*18*). Medical examiner/coroner systems are varied across the United States, ranging from states with only medical examiners, states with only coroners, and states with mixed medical examiner and coroner systems (*18*). Overall, medical examiner systems have larger jurisdictions and operate with more resources than coroner systems. Medical examiner systems are more likely to have electronic death investigation records.

Medical examiner and coroner databases contain predominantly noninfectious disease cases. Therefore, manually reviewing these databases to identify infectious disease cases is inefficient. Developing an automated system that would identify a subset of cases that are likely infectious, and then manually reviewing these cases to identify infectious disease deaths, could reduce the resources that would be necessary to perform infectious disease surveillance. We hypothesized that a computer-assisted search tool could quickly and efficiently detect infectious disease deaths from a computerized medical examiner database, thereby reducing the number of records that would need to be manually reviewed to perform infectious disease surveillance with medical examiner and coroner data.

## Methods

### Case Identification

**Figure 1:**
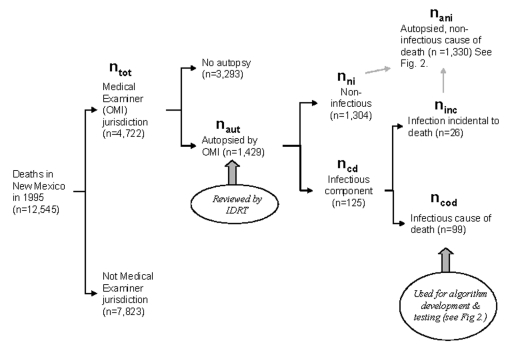
Flow chart for Infectious Disease Death Review Team review and determination of infectious cause of death.

The New Mexico Office of the Medical Investigator (OMI) is a statewide centralized medical examiner agency based at the University of New Mexico School of Medicine. OMI annually performs approximately 90% of the autopsies in New Mexico (*5*). In 1995, New Mexico had a midyear population of 1,682,417; that year, 12,545 deaths occurred in the state (Figure 1) (*19*,*20*). We obtained a database of all deaths (N_tot_ = 4,722) in New Mexico during 1995 that came under the jurisdiction of OMI. From this database, autopsied deaths were identified (n_aut_ = 1,429). A case-patient was defined as a person who died in New Mexico during 1995 who had an infectious disease identified at the time of death and who underwent autopsy by OMI. An expert review panel (Infectious Disease Death Review Team [IDDRT]) reviewed all autopsy records and identified deaths that met the case definition (n_cd_ = 125). On the basis of the findings at autopsy, we further categorized cases as an infectious cause of death (ICOD) (n_cod_ = 99) and infection incidental to death (n_inc_ = 26).

In addition to cause of death (disease or injury that initiates the fatal sequence of events), OMI cases are classified in terms of manner of death (circumstances, i.e., natural, accident, homicide, suicide, or undetermined). Deaths that were considered natural or of undetermined manner comprised 33% (471/1,429) of all OMI autopsies in 1995 and 85% (106/125) of the deaths identified as infectious disease–related by the expert panel. The manner of death was classified as an accident in 39% (561/1,429) of all autopsied persons and in 13% (16/125) of deaths that were identified as infectious disease–related by the expert panel. Homicides and suicides accounted for 28% (395/1,429) of OMI autopsies.

### Expert Review Panel

The IDDRT included specialists in infectious diseases, forensic and clinical pathology, epidemiology, and information technology and was in operation in New Mexico, under the auspices of the OMI, from late 1994 to mid-1996 (*5*,*12*). One forensic pathologist member of the IDDRT routinely reviewed all OMI autopsy records and identified those deaths that were possibly infectious disease–related for review by the expert panel*.*

### Algorithm Development

**Figure 2:**
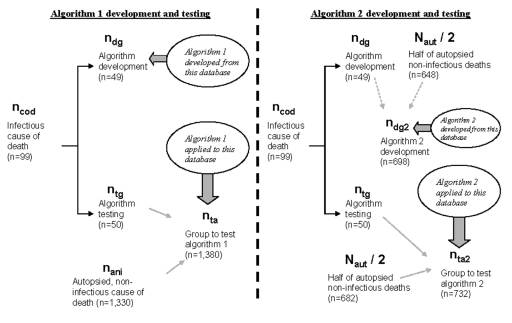
Flow chart for algorithm 1 and 2 development and testing.

We randomly divided ICOD cases into two groups: one group (n_dg_ = 49) was used for algorithm development (i.e., development group); the other group (n_tg_ = 50) was used for algorithm validity testing (i.e., test group). To develop the algorithm, we reviewed the autopsy record for each case in the development group. We developed two separate algorithms based on two separate, but related, datasets (Figure 2). These datasets are described below.

The first algorithm (algorithm 1) was based on data (i.e., truncated dataset) equivalent to information found on the death certificate: demographic variables (e.g., age, sex, and race); cause and manner of death; plus a brief description of the circumstances of death (i.e., a short narrative reported by the death scene investigator). This dataset is referred to as the “truncated’ dataset. We used this dataset because we wanted to evaluate the usefulness of death certificate information for this method because this information may be readily available to persons performing surveillance activities. We developed an algorithm based on the development group of cases (n_dg_ = 49). After the algorithm was developed, we added the test group of cases (n_tg_ = 50) to the noninfectious cases (n_ani_= 1,330) and applied the algorithm to this database (n_ta_= 1,380) to test the algorithm’s ability to detect infectious disease deaths from the truncated database.

The second algorithm (algorithm 2) was developed on the basis of the full text of the pathologists’ dictated autopsy records (i.e., full-text set), which included pathologic observations, pathologic diagnoses, and causes of death. This dataset contains much more detailed information than the truncated dataset. It represents data that may be available from medical examiner offices, in addition to the death certificate. Because we found a low predictive value positive (PVP) and low specificity when algorithm 1 was applied to the full-text dataset, we chose to develop algorithm 2 in a different manner. We randomly selected approximately half (n = 649) of the autopsy-categorized deaths of noninfectious causes (n_ani_ = 1,380) to include in the development of algorithm 2 to reduce the number of false-positive cases the algorithm identified (i.e., cases identified by the algorithm as infectious disease–related but not actually infectious disease-related after expert review). Therefore, the total number of deaths from all manners used for algorithm 2 development was 698 (n_dg2_ = 698), and the total number of deaths used for testing algorithm 2 was 732 (n_ta2_=732). Algorithm development and text searching were performed by using a commercially available software package (AskSam 3.0 Professional, Seaside Software, Inc., Perry, FL).

For developing both algorithms, we manually searched and indexed potential keywords for identifying deaths caused by infectious diseases. From this process, a list of approximately 20 keywords and rules was compiled (i.e., algorithm; see Appendix ). These keywords included entire intact words, words put in a wildcard format (e.g., bacter*, which would flag the words bacterial and bacteremia), and words in a fuzzy search format (e.g., one letter in the word could be wrong, and the word would still be flagged in the record, thus decreasing misclassification caused by misspelling and data entry errors). Rules included searching for words in specific database fields (e.g., *undetermined* in the cause of death field), and proximity rules (e.g., *immune* within two words of *deficiency*).

### Algorithm Implementation and Analysis

The algorithms were applied to the remaining set of records, which included all 1995 OMI autopsied cases except for the development group set of cases (n_trc_ = 1,380 for the truncated dataset; n_ft_ = 732 for the full-text dataset). We applied algorithm 1 to both the truncated and full-text datasets and applied algorithm 2 to the full-text dataset to determine whether an advantage existed in developing an algorithm that used the data from full-text instead of data that could be obtained from death certificates. We determined the sensitivity and PVP of the results by applying the algorithm to this database. Thus, we compared the cases identified from the database by using the algorithm with cases identified by the expert review panel.

## Results

### Algorithm 1: Truncated Dataset

**Figure 3:**
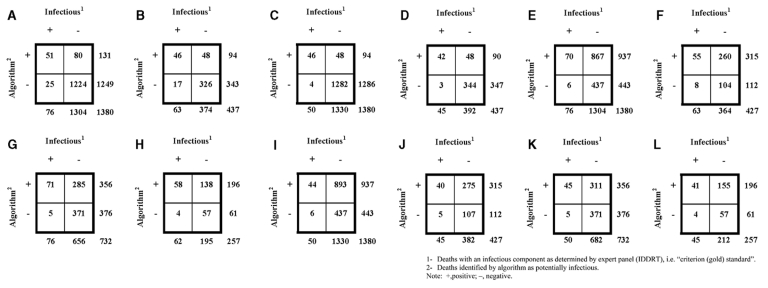
Two-by-two table used to derive predictive positive value.

Algorithm 1 classified 131 (10%) of 1,380 (n_ta)_ autopsied deaths from the truncated dataset as infectious disease–related (Table and Figure 3). Overall sensitivity for identifying both ICOD and incidental infectious diseases was 67% (51/76), and the overall PVP was 39% (51/131). Implementation of the algorithm for surveillance for infectious disease deaths would have resulted in a 91% decrease (131 vs. 1,380) in the number of death records to review. The algorithm identified ICOD cases with a sensitivity of 92% (46/50) and a PVP of 49% (46/94).

**Table T1:** Sensitivity and predictive value positive (PVP) of algorithm 1 and algorithm 2 applied to the truncated and full-text datasets, compared by manner of death and infection as cause of death

	Truncated dataset					Full-text dataset			
	All causes of death		Natural and undetermined causes of death			All causes of death		Natural and undetermined causes of death	
ICOD and incidental infections^a^	Sensitivity	PVP	Sensitivity	PVP		Sensitivity	PVP	Sensitivity	PVP
Algorithm 1	67% (51/76) **1**	39% (51/131) **1**	73% (46/63) **2**	49% (46/94) **2**		92% (70/76) **5**	8% (70/937) **5**	87% (55/63) **6**	17% (55/315) **6**
Algorithm 2	n/a					93% (71/76) **7**	20% (71/356) **7**	94% (58/62) **8**	30% (58/196) **8**
ICOD only**^a^**									
Algorithm 1	92% (46/50) **3**	49% (46/94) **3**	93% (42/45) **4**		45% (42/94)**4**	88% (44/50) **9**	5% (44/937) **9**	89% (40/45)**10**	13% (40/315)**10**
Algorithm 2	n/a					90% (45/50) **11**	13% (45/356) **11**	91% (41/45) **12**	21% (41/196) **12**

^a^ICOD, infectious cause of death. ^b^Number in bold in lower right corner of each cell corresponds to results of 2 x 2 table shown in Figure 3.

Algorithm 1 identified deaths classified as natural or undetermined and with an ICOD and incidental infections from the truncated dataset with a sensitivity of 73% (46/63) and a PVP of 49% (46/94). Implementation of the algorithm for surveillance for infectious disease deaths would have resulted in a 78% decrease (94 vs. 437) in the number of death records to review. The algorithm identified deaths with both a natural or undetermined cause and an ICOD with a sensitivity of 93% (42/45) and a PVP of 45% (42/94).

### Algorithm 1*:* Full-Text Dataset

When algorithm 1 was applied to the full-text dataset, it classified 937 (68%) of 1,380 deaths as infectious disease–related. Sensitivity for accurately detecting all deaths classified as natural or undetermined, for detecting deaths caused by all infections, and for detecting those with an ICOD only, ranged from 88% to 92% (Table)[Table T1]. However, PVP ranged from 5% (for all causes of deaths, ICOD only) to 17% (for those classified as natural or undetermined, classified as ICOD, or identified as incidental infections). Implementation of the algorithm for surveillance of infectious disease deaths would have resulted in a 32% decrease (937 vs. 1,380) in the number of death records to review for all causes of death, and a 26% decrease (315 vs. 427) for only natural and undetermined causes of death.

### Algorithm 2: Full-Text Dataset

Algorithm 2 (developed on the basis of the full-text dataset, which included panel-confirmed infectious disease deaths and 50% of the noninfectious disease-related deaths) was applied to the full-text dataset only. The sensitivity of the algorithm to identify infectious disease–related deaths ranged from 90% (all deaths; ICOD only) to 94% (natural or undetermined; ICOD or incidental infections). PVP ranged from 13% (all deaths; ICOD only) to 30% (natural or undetermined; ICOD or incidental infections). Implementation of the algorithm for surveillance of infectious disease deaths would have reduced by 51% (356 vs. 732) the number of death records to review for deaths from all causes and reduced by 24% the records to review (196 vs. 257) of those deaths categorized as having natural and undetermined causes.

## Discussion

A simple computer text search tool (i.e., algorithm) can efficiently detect infectious disease deaths from a medical examiner’s database, demonstrating that this technique can be an essential tool in the surveillance for infectious diseases of public health importance. Medical examiners are a critical public health resource for fatal infectious disease surveillance (*5*,*12*). Ideal surveillance at medical examiner offices would include active case finding, as has been implemented in a pilot program in New Mexico funded by the Centers for Disease Control and Prevention (*21*). Because infectious disease surveillance that uses medical examiner data does not occur in a standardized manner, a computer text search tool could be implemented by jurisdictions that otherwise might not have the resources to perform these activities. Implementation of this technique nationally would require large-scale development of electronic databases in medical examiner’s offices and subsequent incorporation of this surveillance tool into routine activities. However, this method is also applicable to surveillance for fatal infectious diseases and other conditions at all medical facilities that collect text-based clinical data, such as emergency departments, inpatient and outpatient settings, and poison control centers.

The sensitivity of the algorithm varied depending on whether it was applied to dictated autopsy records, including all pathologic diagnoses, or to a truncated dataset containing records equivalent to that found on a death certificate (i.e., basic demographic information and causes of death). The expense of improved sensitivity is that more records must be reviewed because of false-positive results. Algorithm application on the truncated dataset achieved a sensitivity similar to that achieved with the full-text dataset, and with a higher PVP, for deaths in which an infection is a cause of death rather than incidental to the death. In addition, for the full-text dataset, sensitivity and PVP were not substantially compromised by including in the search, infections incidental to the cause of death. Clearly, incidental infections are found among persons who die from homicide, suicide, or accidents. Recognizing incidental infections could be critical for surveillance systems designed to identify chronic infections such as tuberculosis and hepatitis C. Sensitivity was not compromised by including deaths from all causes rather than deaths from natural and undetermined causes. PVP was increased somewhat by restricting the search to deaths classified as natural and deaths classified as undetermined. Still, medical examiner–based infectious disease surveillance could effectively use complete data sets rather than data subsets.

 This study was possible because of the findings from the expert review of infectious disease deaths which could be compared with data generated by the algorithm. This panel reviewed records from approximately 90% of all autopsies that occurred in New Mexico in 1 year and, of these deaths, likely ascertained all OMI cases with an infectious disease component. However, the implementation of such a review process using manually retrieved cases might not be feasible in medical examiner jurisdictions with limited resources and a large case volume. A computerized algorithm could allow for surveillance in settings where it otherwise might be impossible. Minimal resources would be required to run the necessary software and review results on a daily basis. Required software is inexpensive, and running the algorithm would require minutes per day. Staff to interpret the results is the main resource that would be required. These results demonstrated a substantial decrease in the number of records that would need to be reviewed with algorithm implementation, compared with those required by manual review alone.

In the future, search algorithms could be used in settings where the records from several medicolegal jurisdictions (e.g., a region consisting of more than one city, county, or state) are combined. As outbreaks of infectious diseases, whether naturally occurring or bioterrorism-related, might span jurisdictional boundaries, computerized records could be compiled from different areas and an algorithm applied to seek patterns or clusters of deaths of one type during a given period. To carry out such cross-regional surveillance, standardized platforms of data collection that would allow for data aggregation are required. Similar algorithms could be used as permanent or temporary surveillance systems designed to detect bioterrorism-related deaths or particular outbreaks. In addition, these algorithms could be modified to evaluate notifiable disease reporting in a jurisdiction. Artificial intelligence techniques could be used to improve algorithm accuracy. Artificial intelligence technology could take algorithm development from rules derived from human testing of specific terms and conditions (as performed in this study), to algorithm development with computer intelligence techniques that develop computer-derived rules. Finally, algorithms could be developed that would identify deaths caused by noninfectious conditions of public health importance.

This study documents a first step in using computer-assisted text search tools to implement and improve infectious disease surveillance with medical examiner data. Research on computerized disease identification through medical information is in the early stages (*22*). Improvements in the algorithm, in algorithm development techniques (such as improving search terms), and in applying algorithms in more diverse ways could enhance the accuracy and usefulness of this method. Currently, increased national and international attention is focused on infectious disease surveillance. Novel surveillance strategies that provide timely and detailed data will likely become important adjuncts to traditional surveillance for fatal infectious diseases.

## Supplementary Material

Appendix Rule Set Used for Algorithm 1
