# Supplementary material for: Fatal Infectious Disease Surveillance in a Medical Examiner Database
Source: Emerg Infect Dis. 2004 Jan;10(1):48–53. doi: 10.3201/eid1001.020764 (PMC3322763; doi:10.3201/eid1001.020764)
Supplement: Appendix — Rule Set Used for Algorithm 1 [file 02-0764_app-s1.pdf]

Publisher: CDC; Journal: Emerging Infectious Diseases
Article Type: Research; Volume: 10; Issue: 1; Year: 2004; Article ID: 02-0764
DOI: 10.3201/eid1001.020764; TOC Head: Research

Appendix. Rule Set Used for Algorithm 1

Entire words[a]	Wildcard words[b]	Fuzzy words[c]	Words in specific fields[d]	Proximity searches[e]
Viral	*itis	Hemorrhagic	*Undetermined* in cause of death field	*immune* within 2 words on either side of *syndrome*
AIDS	septic*	Thrombocytopenia	*Unknown* in cause of death field	
Sepsis	necro*	Hemolytic		
	pneumo	Tuberculosis		
	gangren*			
	bacter*			
	infect*			
	mening*			
	absces*			

[a]Entire words are searches for the word as written.
[b]Wildcard words are searches for the string of letters as written, with the asterisk representing any letter or letters. For example, *gangren** would represent gangrene and gangrenous.
[c]Fuzzy words are searches for the word as written, but one letter may be misspelled, missing, inserted, or transposed, and the search will still identify that word. For example, a fuzzy search for *hemorrhagic* would identify hemorrhagic, haemorrhagic, and hemorhragic.
[d]Words in specific fields are searches for the words as written in specific database fields, in this case the cause of death fields.
[e]Proximity searches are searches for words in a given proximity to other words. For example, *immune* within 2 words on either side of *syndrome* would identify immune syndrome, immune deficiency syndrome, and syndrome of immune deficiency.
